# Supplementary material for: Quantum simulation of particle creation in curved space-time
Source: PLoS One. 2020 Mar 6;15(3):e0229382. doi: 10.1371/journal.pone.0229382 (PMC7059940; doi:10.1371/journal.pone.0229382)
Supplement: S1 Appendix — (PDF) [file pone.0229382.s001.pdf]

S1 Appendix. Approximation of the SAW speed.

For convenience, the 2DEG density is denoted by n in the following. In principle, an expression for the SAW speed $c(x)$ in terms of n can be obtained from the implicit equation Eq 20, which gives an *algebraic* relation between the SAW speed and the 2DEG density. Consequently, $c(x)$ only depends *locally* on $n(x)$ and $\partial_x n(x)$: As $n(x)$ and $\partial_x n(x)$ only change at the gate's edge on a length scale κ_s^{-1}, $c(x)$ changes on a length scale κ_s^{-1}, too. This can be also seen as follows: $c(x) = f(n, \partial_x n)$ with a particular function f, and derivation of this equation with respect to x gives

$$\frac{\partial c}{\partial x} = \frac{\partial f}{\partial n}\frac{\partial n}{\partial x} + \frac{\partial f}{\partial\left(\partial_x n\right)}\frac{\partial^2 n}{\partial x^2}. \tag{S1}$$

Expanding $n(x)$ (see Eq 24) in a Taylor series around the gate's edge $x = 0$ up to third order and solving for the roots, one obtains a value of approximately $4\kappa_s^{-1}$ for the size of the region, where $n(x)$ changes from 0 to its maximum value. Outside this region $\partial_x n$ and $\partial_x^2 n$ vanish. According to Eq S1, the SAW speed consequently only changes around the gate's edge, too. As a result, $c(x)$ can be approximated as follows: In the regions inside and outside the gate with a distance to the gate's edge greater than $2\kappa_s^{-1}$, the SAW speed is assumed to be constant, with values given by letting $n \to \infty$ and $n \to 0$, respectively, and $\partial_x n \to 0$ in Eq 20, giving $c = c_0 = \sqrt{d/\rho}$ and $c = c_0\sqrt{1 + K^2} \approx c_0\left(1 + \frac{1}{2}K^2\right)$, respectively, where a small piezoelectric coupling constant $K^2 = e^2/(\varepsilon d) \ll 1$ is assumed, which is valid for nearly all piezoelectric materials [32]. In the remaining region of size $4\kappa_s^{-1}$ around the gate's edge, the SAW speed can be assumed to adjust linearly between these two values.
